# Supplementary figures and images for: Forest Tree Associated Bacterial Diffusible and Volatile Organic Compounds against Various Phytopathogenic Fungi
Source: Microorganisms. 2020 Apr 18;8(4):590. doi: 10.3390/microorganisms8040590 (PMC7232321; doi:10.3390/microorganisms8040590)

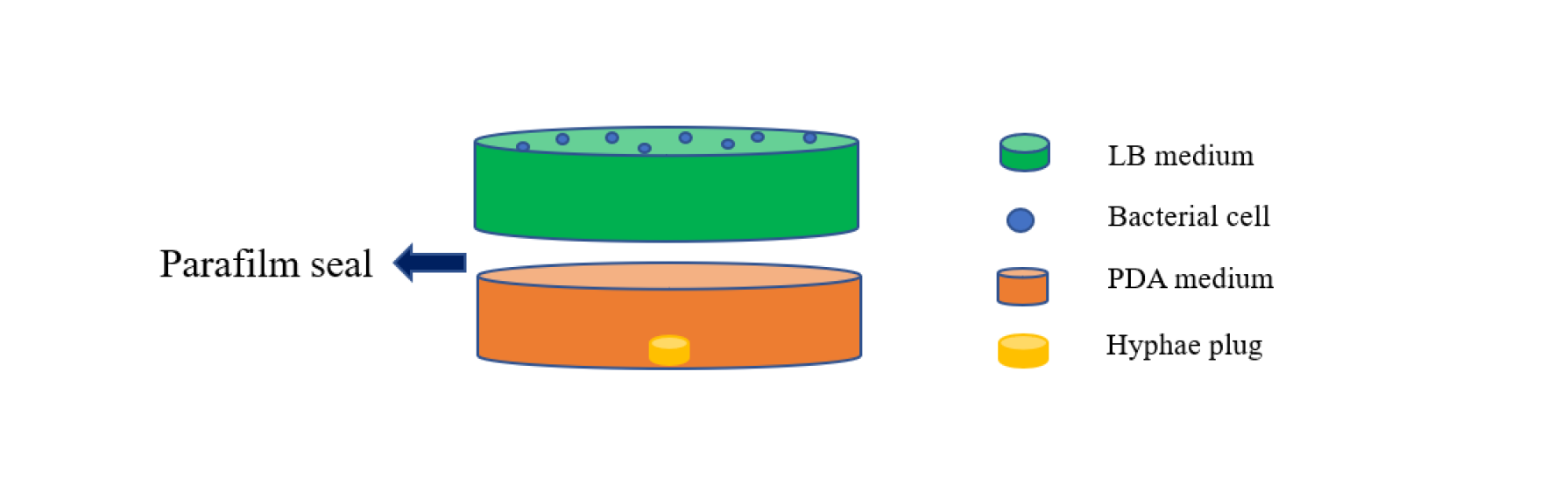

Supplement: Supplementary file 1 [file microorganisms-08-00590-s001.zip › microorganisms-766843-supplementary.tif]
